# Deliberation and confidence change

**DOI:** 10.1007/s11229-022-03584-3

**Published:** 2022-02-25

**Authors:** Nora Heinzelmann, Stephan Hartmann

**Affiliations:** 1 Institute of Philosophy, Friedrich Alexander University of Erlangen-Nuremberg, Bismarckstrasse 1, 91054 Erlangen, Germany; 2 Munich Center for Mathematical Philosophy, Ludwig Maximilian University, Geschwister-Scholl-Platz 1, 80539 Munich, Germany

**Keywords:** Deliberation, Credence, Confidence, Bayesian updating, Social epistemology

## Abstract

We argue that social deliberation may increase an agent’s confidence and credence under certain circumstances. An agent considers a proposition H and assigns a probability to it. However, she is not fully confident that she herself is reliable in this assignment. She then endorses H during deliberation with another person, expecting him to raise serious objections. To her surprise, however, the other person does not raise any objections to H. How should her attitudes toward H change? It seems plausible that she should (i) increase the credence she assigns to H and, at the same time, (ii) increase the reliability she assigns to herself concerning H (i.e. her confidence). A Bayesian model helps us to investigate under what conditions, if any, this is rational.

## Introduction

Suppose that you read a newspaper article discussing the claim that masks lower the risk of coronavirus transmission. You believe that it is true but you do not absolutely believe it. Your credence is, say, 0.7. That is, you assign a probability of 0.7 to the proposition designated by “masks lower the risk of coronavirus transmission.”

The article first quotes an influential politician who says that he is “all for masks”, indicating that he and you would agree that masks lower the risk of transmission. Perhaps he would even assign the same credence to it: 0.7. But you do not believe that the politician is very reliable^1^ in assigning this credence — previously, he denied the effectiveness of masks, and other measures he claimed to be effective turned out not to be.

We can conceptualise the reliability assigned to a person for a given proposition as a number ranging from 0 (completely unreliable) to 1 (completely reliable). A completely unreliable person would make entirely random reports. Their statements would never be related to the truth. Even if they were true, they would be true merely by coincidence. Others could never rely on what they said. To such a person, we could assign a reliability of 0. But they are a hypothetical person; most people of flesh and blood are not that unreliable, not even your erratic politician. Imagine that you assign 0.2 to them regarding the claim that masks lower the risk of coronavirus transmission.

Next, the article quotes a distinguished expert who has made a career in epidemiology. This person, too, states that masks lower the risk of coronavirus transmission. So the politician, the epidemiologist, and you all tend to agree that the claim is true. Perhaps you would even assign the same credence to it: 0.7. But the epidemiologists seems much more reliable to you than the politician. A *completely* reliable person would be a truth teller: their statements would always be true, and others could rely on them entirely. To such an ideal person, we could assign a reliability of 1. But no person of flesh and blood is *that* reliable, not even your epidemiologist, although she comes close. You assign to her a reliability of, say, 0.9 regarding the claim in question. Note that this assignment is proposition-specific: you would probably not assign the same reliability to the epidemiologist regarding a claim about, e. g., the evolutionary underpinnings of sexual dimorphism in the Argiope bruennichi species.

What reliability, though, do you assign to yourself? Presumably, you regard yourself as somewhat more reliable than the politician but also as less reliable than the epidemiologist. You are not a truth teller but you are not entirely erratic either. Perhaps you assign a reliability of 0.5 to yourself regarding the claim that masks prevent coronavirus transmission.^2^ We could interpret this self-assigned reliability as a figure indicating that you are in-between a truth teller and an entirely erratic person. Just as in the case of third-party ascriptions, we can conceptualise the self-ascribed reliability as a number ranging from 0 to 1. The greater the number, the more reliable an agent considers herself. If she regards herself as completely reliable, we may conceptualise this as a self-ascription of 1. If she regards herself as completely unreliable, we may conceptualise this as a self-ascription of 0. In our example, you regard yourself as in-between of those two extremes; you assign a reliability of 0.5 to yourself.

Just as for other agents, reliability self-assignments like this are specific for the claim in question. You would assign a much higherreliability to yourself concerning, say, claims about your favourite colour, and presumably a much lowerreliability concerning claims about the evolutionary underpinnings of sexual dimorphism in the Argiope bruennichi species.

Here we borrow a term from the behavioural sciences to refer to an agent’s self-assigned reliability regarding a proposition H: “confidence”. In the sciences, confidence is generally described as the “feeling of knowing” that H or more specifically as the probability of being correct in a prior choice, decision, or claim, as estimated by the agent (Fleming 2010; Martino 2013; Pouget et al. 2016; Navajas 2018). The probability thus ranges over a random variable that can take two values, correct or incorrect. In a typical study, a participant would first be asked to complete a task, e. g., to estimate the likelihood that masks lower the risk of coronavirus transmission. Their confidence is then measured by asking them to indicate on a scale from 0% to 100% the probability that the estimate they have just reported is correct. Confidence has been identified as a key factor in a range of domains, such as perception (Navajas 2017), value judgements (Folke 2016), or social cooperation (Bahrami 2010).

Besides borrowing the term “confidence”from the behavioural sciences, we also largely follow its usage in modeling confidence as a probability over a binary variable. However, we specify this variable further as the agent’s self-assigned reliability, in analogy to the third-person testimony case. For example, just as a witness may report a credence of 0.7 and we may assign to them a reliability of 0.2 concerning this report, we ourselves may report the very same credence but assign to ourselves a reliability of 0.5 concerning this report.^3^

Our conception of confidence thus differs from that of authors who use “confidence”, “credence”, or “degree of belief” synonymously (Lasonen-Aarnio 2013), or who take confidence as a betting disposition or affective state that is explained or determined by credence (Christensen 2009; Frances and Matheson 2019). It might turn out that confidence is related or can even be reduced to resistance to revision (Levi 1980), credal resilience (Skyrms 1977; Egan and Elga 2005), higher-order uncertainty (Dorst 2019, 2020), or evidential weight (Nance 2008; Joyce 2005), yet these questions are not our concern in the present paper.

In this paper, we focus on the following issue: When you put a proposition to the test of critique and objection and fail to encounter them, how ought your confidence and credence regarding this proposition change? We address this question in the next section.

## Deliberation

Let us assume that you show the newspaper article to a friend. Regarding the claim about masks, you assign a reliability of 0.7 to your friend. That is, you think that she is not as reliable as the epidemiologist but somewhat more reliable than you yourself. Unlike yourself, she has a PhD in medicine and works as a physician in a hospital that treats coronavirus patients. When the two of you begin deliberation, you expect her to raise substantial objections to the claim that masks lower the risk of coronavirus transmission. However well researched, the article is merely a news item, presumably fails to mention some important caveats, and does not present and assess the evidence as well as your friend does. You do not know what her concerns will be, even less whether they are the very same ones you have already considered. Your friend might even side with you on the issue after having raised—and rebutted—some objections.

You begin the deliberation by publicly stating the claim you are entertaining: “masks lower the risk of coronavirus transmission.” For the sake of conversation, then, you endorse the proposition. At the same time, you harbour doubts about what you just said. Will your friend respond with a thorough rebuttal? The two of you deliberate about the claim, the article and the evidence and quotes it provides, as expected. However, to your surprise, you begin to realise that your expectation does not become reality. When deliberation ends, you find that your friend did not provide new and serious objections to your claim. How should this experience affect your credence and confidence?

Note that, in this paper, we are not interested in how an agent ought to respond to peer disagreement (Frances and Matheson 2019). We target the question of whether and how an agent ought to rationally update their credence and confidence in light of the fact that an interlocutor does not raise (novel) objections, regardless of whether or not they disagree and regardless of whether or not they are a peer (we briefly discuss the role of experts and peers below in Sect. 3). Furthermore, our question is closely related but not identical to the question of how we ought to update our credence and confidence once we learn someone else’s credence and confidence (Easwaran et al. 2016). In our case, you do not need to learn what your interlocutor’s credence is—you merely find that they fail to raise objections to your view. How, then, should the exposure to possible objections during deliberation affect the agent’s confidence and credence? We turn to a Bayesian model to answer this question.

## A Bayesian model

It is non-trivial to construct a Bayesian model on how a rational agent should change her confidence once new evidence from deliberation comes in (in this case the evidence is the observation that the consulted friend does not raise new objections). For one thing, standard Bayesian models of testimony assume that the reasoning agent is not identical with the person who provides the respective testimony. In such cases, the reasoner assigns a prior to the hypothesis under consideration and a reliability to the witness. But can one also assign a reliability (or confidence) to oneself? And how can one model the updating of one’s own confidence?

We propose to use a slightly extended and modified version of the model of testimony introduced in Bovens and Hartmann (2003).^4^ This model specifies how a rational agent updates her credence when receiving a witness report. The agent updates her credence on the basis of the testimony report on the one hand and on the presumed reliability of that report on the other hand.

Our modifications of this model here are twofold: First, we replace the reliability (which one assigns to others) with the *confidence* (that one assigns to oneself).^5^ Second, we replace the testimony report with the *endorsement* of the agent in a situation of deliberation. Endorsement is a doxastic attitude of commitment towards a proposition but differs from belief (cf. Fleisher 2018; Cohen 1992). Importantly, the agent can endorse a proposition even if their respective credence and confidence are low. In science, a researcher may rationally endorse a speculative hypothesis on the basis of which he conducts experiments; in social deliberation, a person may endorse a claim even though she is not fully convinced of it. Whilst it is irrational to endorse a claim one knows to be false, it is rationally permissible to endorse a proposition that is unlikely to be true. Furthermore, we assume that the agent endorses H with a certain probability which depends on her confidence as well as on the truth or falsity of the proposition in question. Lastly, note that our model does not specify the psychological mechanism of endorsement; what is crucial is that endorsement influences credence as well as confidence (similar to the mechanism generating the testimony report in the Bovens and Hartmann model).

### The baseline model

Let us now become more precise. To do so, we need to specify the variables we consider and how they relate. First, we assume that the agent entertains the following four propositional variables in the situation at hand: *H*, *E*, *C*, and *O*. *H* has the values H: “The proposition in question is true” and $\lnot$H: “The proposition in question is false”, *E* has the values E: “I endorse the proposition” and $\lnot$E: “I do not endorse the proposition”, *C* has the values C: “I am (fully) confident about the proposition" and $\lnot$C: “I am not confident about the proposition", and *O* has the values O: “The interlocutor provides serious objections to the proposition” and $\lnot$O: “The interlocutor does not provide serious objections to the proposition”. In the present situation, the agent is uncertain about the values of the propositional variables *H*, *E*, *C*, and *O*, and therefore specifies a probability distribution *P* over them.

**Fig. 1:**
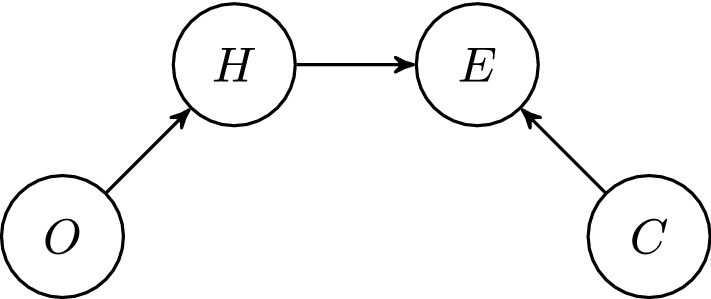
The Bayesian network representing the epistemic situation of the agent

Second, the Bayesian network in Fig. 1 represents the probabilistic relations that hold between the four propositional variables. It assumes that (i) *O* and *C* are root nodes (and hence independent of each other), (ii) *H* and *C* are independent of each other, and (iii) through the endorsement E (once it is made) *H* correlates with *C*. Strictly speaking, then, the agent’s confidence is her self-assigned reliability concerning her endorsement of a proposition and, where no endorsement is made, a hypothetical endorsement. This corresponds to the witness reliability regarding actual or hypothetical testimony reports in the Bovens and Hartmann model. However, we can for the sake of convenience speak more loosely of the reliability concerning the proposition. The model thus assumes strict separation of the credence of the proposition and the confidence in the corresponding endorsement. However, once the endorsement is made, the value of *O* (and, in turn, the value of *H*) becomes relevant for *C* (as we will show below).

We now fix the prior probabilities of the root nodes,

1 $$\begin{aligned} P(\mathrm{O}) = o, \quad P(\mathrm{C}) = c, \end{aligned}$$

and the conditional probabilities of the child node *H*, given the values of its parent:

2 $$\begin{aligned} P(\mathrm{H \vert O}) = p, \quad P(\mathrm{H \vert \lnot O}) = q \end{aligned}$$

We assume that a rational agent is at least somewhat receptive towards the other person with whom they converse. They are ready to adjust their credence in response to the other person’s objections. From the agent’s perspective, the other person could be an epistemic peer, an expert, or neither. What is crucial is that the agent’s probability ascriptions about *O* must be sensitive to the fact that the interlocutor raises objections (or not). Plausibly, the more an agent regards the other person as an expert, the higher will be the value she ascribes to *q*, and the smaller will be the value she ascribes to *p*.

As the interlocutor’s expected objections constitute evidence against H (Eva and Hartmann 2018), we require that

3 $$\begin{aligned} p < q. \end{aligned}$$

Note that *p* and *q* need not add up to 1, although $P(\mathrm{O})$ and $P(\mathrm{\lnot O})$ presumably do.

Finally, we fix the conditional probabilities of *E*, given the values of its parents:

4 $$\begin{aligned} P(\mathrm{E \vert H, C}) = 1&,&\quad P(\mathrm{E \vert \lnot H, C}) = 0 \nonumber \\ P(\mathrm{E \vert H, \lnot C}) = a&,&\quad P(\mathrm{E \vert \lnot H, \lnot C}) = a \end{aligned}$$

Here we use a modification of the model proposed by Bovens and Hartmann (2003, : ch. 3) which assumes that the agent is either fully confident or not confident.^6^ If the agent is (fully) confident, then she endorses the proposition in question in deliberation if *H* is true, and she does not endorse the proposition in question if *H* is false.

If the agent is not confident, then she endorses the proposition in question during deliberation with a certain probability *a* independently of whether *H* is true or not. Here *a* is a measure of the agent-specific likelihood to endorse a proposition during deliberation despite lacking confidence. This likelihood is similar to a character trait. For instance, an agent with a high *a* indiscriminately endorses any proposition even when they are not at all confident. Our model requires that $a < P(\mathrm{H})$, that is, the agent’s likelihood to endorse the proposition in question when lacking confidence must be lower than the prior probability ascribed to the proposition in question. In Humean words, the agent is required to proportion this probability to her likelihood of endorsement.

With this, we can prove two theorems (the detailed proofs are in the appendix):

#### Theorem 1

Consider the Bayesian network from Fig. 1 with the prior probability distribution *P* as specified in Eqs. (1), (2) and (4). Then condition (3) implies that $P(\mathrm{H\vert E, \lnot O})> P(\mathrm{H\vert E}) > P(\mathrm{H})$.

This is plausible:

1. Once the agent endorses a proposition, e. g., once she makes a public announcement to the effect that H during deliberation, her credence in H increases.
2. Once the agent also learns that the other person does not provide objections as expected, her credence in H increases one more time.

#### Theorem 2

Consider the Bayesian network from Fig. 1 with the prior probability distribution *P* as specified in Eqs. (1), (2) and (4). Then condition (3) and $a < P(\mathrm{H})$ imply that $P(\mathrm{C\vert E, \lnot O})> P(\mathrm{C\vert E}) > P(\mathrm{C})$.

This is plausible:

1. Once the agent endorses a proposition, i. e., once she makes, e. g., a public announcement to the effect that H during deliberation, the confidence in herself concerning H increases (provided that *a* is sufficiently small).
2. Once the agent also learns that the other person does not provide objections as expected, the confidence increases one more time (provided, again, that *a* is sufficiently small).

It is interesting to note that a different ordering of $P(\mathrm{C\vert E, \lnot O})$, $P(\mathrm{C\vert E})$ and $P(\mathrm{C})$ obtains if $a \ge P(\mathrm{H})$ (or even $a \ge q$). For details, see the proof of Theorem 2. The explanation of this phenomenon is analogous to the corresponding explanation given in Bovens and Hartmann (2003, ch. 3.2) for the testimony case.

### Relaxing an idealization

So far we have assumed that the propositional variables *C* and *O* are independent. In other words, we have assumed that how confident I am does not affect my expectations about objections from an interlocutor, or vice versa. However, this is an idealization as it is plausible that *C* and *O* are negatively correlated. That is, if I have a low confidence, I am more likely to expect serious objections than if I have a high confidence. Therefore, in this section we present a more complex model which assumes that *C* and *O* are negatively correlated. We shall obtain similar results as before, i. e., according to our revised model it will turn out that it can be rational to increase both confidence and credence.

**Fig. 2:**
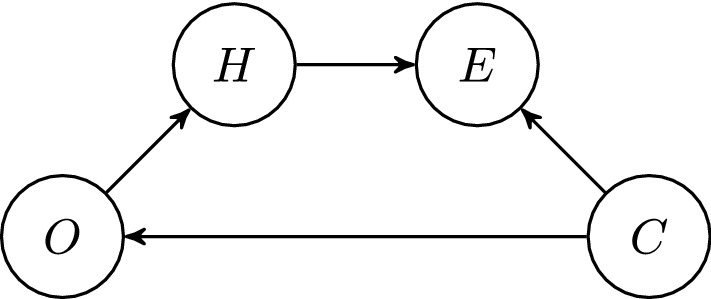
The new Bayesian network representing the epistemic situation of the agent

The Bayesian network in Fig. 2 models the situation when confidence negatively correlates with expectations of objection. We set

5 $$\begin{aligned} P(\mathrm{C}) = c \end{aligned}$$

and

6 $$\begin{aligned} P(\mathrm{O\vert C}) = \alpha \quad , \quad P(\mathrm{O\vert \lnot C}) = \beta . \end{aligned}$$

The condition

7 $$\begin{aligned} \alpha < \beta \end{aligned}$$

models the intuition that it is more likely that one expects serious objections if one has a low confidence than if one has a high confidence.^7^

With this, we can prove two theorems (see the appendix for the detailed proofs):

#### Theorem 3

Consider the Bayesian network from Fig. 2 with the prior probability distribution *P* as specified in Eqs. (2), (4), (5) and (6). Then conditions (3) and (7) imply that $P(\mathrm{H\vert E, \lnot O})> P(\mathrm{H\vert E}) > P(\mathrm{H})$.

#### Theorem 4

Consider the Bayesian network from Fig. 2 with the prior probability distribution *P* as specified in Eqs. (2), (4), (5) and (6). Then conditions (3), (7) and $a < P(\mathrm{H \vert C})$ imply that $P(\mathrm{C\vert E, \lnot O})> P(\mathrm{C\vert E}) > P(\mathrm{C})$.

That is, the results of Theorems 1 and 2 basically also hold if *C* and *O* are negatively correlated. The only difference of our more complex model is that the condition $a < P(\mathrm{H})$ in Theorem 2 has to be replaced by $a < P(\mathrm{H \vert C})$ in Theorem 4. On the assumption that confidence and expectations of objection correlate negatively, then, it remains rational to increase one’s confidence and credence when failing to meet objections to a proposition one open-mindedly endorses in conversation.

### Informal interpretation

This section interprets the results of our proofs informally. Let us consider credence first. Credence is the probability the agent assigns to a proposition. In our example, your initial credence is 0.7. It seems unlikely that the agent ought to lower their credence when objections are expected but not raised. Perhaps, then, it is rational to retain one’s credence. After all, the view under discussion has not met new challenges, so there seems to be no reason to update it at all. However, we suggest that in the case of interest, it may be rational, given plausible assumptions, to *increase* one’s credence in a proposition. There are at least two reasons for this. The first reason is that in conversation the agent endorses the proposition in question. That is, they commit to it, even though they do not fully believe in it. In our example, this happens when you publicly declare that masks lower the risk of coronavirus transmission. You thus accept the proposition as a premise in your reasoning and argumentation. Apparently, agents seem to act in accordance with this reason in real life. For, it has been shown empirically that endorsing a proposition increases the agent’s credence (Schwardmann et al. 2019), (cf. Mercier and Sperber 2011; Heinzelmann et al. 2021).

Note that the rational constraints (specified in 4 of our model) prevent the agent from irrational bootstrapping (Weisberg 2012).^8^ Bootstrapping would occur if the agent, merely by playfully endorsing a proposition, could thereby generate a reason to increase their credence, as it were. However, rational endorsement of a proposition is not playful endorsement, it is constrained in a number of ways. For one thing, a fully rational and fully confident agent does not endorse a proposition they believe to be false. Consequently such an agent could not generate a high credence by bootstrapping.

In our example, although you endorse the proposition during deliberation, you remain open to abandoning it when met with substantial objection from your interlocutor. But then no new objection is made during deliberation. This provides you with an additional reason for increasing your credence. For one thing, the mere fact that an agent has not yet come across a piece of testimony that F is evidence that not-F, and conversely, failure to encounter testimony that not-F provides the agent with a reason to believe that F (Goldberg 2011 cf.Mulligan 2019). Relatedly, lacking an objection to H may constitute a reason for H because lacking a reason for F may constitute a reason against F (Eva and Hartmann 2018). More generally, as our model implies, a proposition may gain support from deliberation when it is not met with opposition: in our example, you had put a proposition to the test of argumentative falsification, and it was not falsified. You cannot be certain, of course, that no killer objections to the claim exist. But so far you have not encountered them even though you were expecting them and prepared to retract the proposition in response. Hence, it seems rational that credence may rise when an interlocutor fails to raise new objections during deliberation.

Let us consider confidence next. In our example, you are initially 50% confident about the proposition you endorsed. How should this assignment have changed after deliberation? It seems that there are three options. A first possibility is that, even if you do not change your credence in the proposition, you ought to lower your confidence. But this seems unlikely; not encountering objections does not seem to be a good reason for becoming less confident in oneself. A second possibility is that your confidence should remain the same. After all, the mere fact that someone fails to object to your view may license you to remain as confident as you are. Here, we suggest that it may be rational, given plausible assumptions and under certain circumstances, to *increase* one’s confidence concerning a proposition after exposing it to the possibility of objection.

There are at least two reasons analogous to the ones given for increased credence. First, for the sake of argument, you endorse the claim put up for discussion. As a consequence you become more confident. Second, when no objection is made during deliberation, you have a new reason for increasing your confidence. For, you have expected but not encountered objections to the proposition that masks lower the risk of coronavirus transmission. This licenses you to be more confident about your view on the matter.

In short, then, when open-mindedly putting a proposition to the test of social deliberation, emerging from this encounter with increased credence and confidence is rational when the proposition is not met with objection.

## Conclusion

We have explained why an agent may increase her confidence and credence after social deliberation. Furthermore, we have argued and showed that it is rational to do so when the agent expects the interlocutor to raise objections, is ready to adjust her credence and confidence accordingly, yet is not confronted with objections as expected. In other words, we have provided arguments and proofs for Mill’s claim that a rational agent, when open-mindedly endorsing a proposition in social deliberation should increase both their confidence and credence in this proposition when it is not met with objection.

## A Proof of Theorem 1

We consider the Bayesian network in Fig. 1 and use the machinery of Bayesian networks as explained in, e.g., Hartmann (2020). With this, it is easy to see that $P(\mathrm{H}) = o \, p + \overline{o}\, q =: h$, where we have used the notation $\overline{x} := 1-x$ which we will also use below. Note that the above expression for $P(\mathrm{H})$ and condition (3) imply that $p< h < q$. Next, we use the *product rule* and calculate $P(\mathrm{H \vert E}) = h\, (c + a\, \overline{c})/(h \, c + a\, \overline{c})$ and $P(\mathrm{H \vert E, \lnot O}) = q \,(c + a\, \overline{c})/(q \, c + a \, \overline{c})$. As $P(\mathrm{H \vert E})$ is strictly monotonically increasing in *h*, condition (3) implies that $P(\mathrm{H \vert E, \lnot O}) > P(\mathrm{H \vert E})$. Finally, we find that $P(\mathrm{H \vert E}) - P(\mathrm{H}) = c \, h \, \overline{h}/(h \, c + a \, \overline{c}) > 0$. Hence, $P(\mathrm{H \vert E}) > P(\mathrm{H})$. This completes the proof. $\square$

## B Proof of Theorem 2

Proceeding as in the proof of Theorem 1, we calculate $P(\mathrm{C \vert E}) = h\, c/(h \, c + a\, \overline{c})$ and $P(\mathrm{C \vert E, \lnot O}) = q \, c /(q \, c + a \, \overline{c})$. Note that $P(\mathrm{C \vert E})$ is strictly monotonically increasing in *h*. Hence, $p< h < q$ implies that $P(\mathrm{C \vert E, \lnot O}) > P(\mathrm{C \vert E})$. Finally, we calculate $P(\mathrm{C \vert E}) - P(\mathrm{C}) = c \, \overline{c}\, (h-a)/(h \, c + a \, \overline{c})$. Hence, $P(\mathrm{C \vert E}) > P(\mathrm{C})$ if $a < h$. This completes the proof.

As a consequence of the results reported in this proof, we note that different orderings obtain if $a > h$ (and all other assumptions are left unchanged). We distinguish two cases: (i) $h< a < q$ implies that $P(\mathrm{C \vert E, \lnot O})> P(\mathrm{C}) > P(\mathrm{C \vert E})$ and (ii) $h< q < a$ implies that $P(\mathrm{C})> P(\mathrm{C \vert E, \lnot O}) > P(\mathrm{C \vert E})$. $\square$

## C Proof of Theorem 3

We consider the Bayesian network in Fig. 2 and define the likelihoods $l_\alpha := \alpha \, p + \overline{\alpha }\, q$ and $l_\beta := \beta \, p + \overline{\beta }\, q$. Then we calculate $P(\mathrm{E}) = l_\alpha \, c + a \, \overline{c}$ and $P(\mathrm{H}) = l_\alpha \, c + l_\beta \, \overline{c}$. Analogously, we obtain

$$\begin{aligned} P(\mathrm{H \vert E})= & {} \frac{l_\alpha \, c + a \, l_\beta \, \overline{c}}{l_\alpha \, c + a \, \overline{c}} \\ P(\mathrm{H \vert E, \lnot O})= & {} \frac{\overline{\alpha }\, c + \overline{\beta }\, a \, \overline{c}}{\overline{\alpha }\, q \, c + \overline{\beta }\, a \, \overline{c}} \cdot q. \end{aligned}$$

Next, we define $\Delta _1 := P(\mathrm{H \vert E}) - P(\mathrm{H})$ and $\Delta _2 : = P(\mathrm{H \vert E, \lnot O}) - P(\mathrm{H \vert E})$ and obtain:

$$\begin{aligned} \Delta _1= & {} \frac{l_{\alpha } \, \overline{l_{\alpha }} \, c + (a \, \overline{l_{\alpha }} \, l_{\beta } + \overline{a} \, l_{\alpha } \, \overline{l_{\beta }}) \, \overline{c}}{l_\alpha \, c + a \, \overline{c}} \cdot c \\ \Delta _2= & {} \frac{\left[ (\alpha \, \overline{q}+ \beta \, q) \, c + \beta \, a \, \overline{c}\right] \, \overline{\beta }\, (q-p) + (\beta -\alpha ) \, q \, \overline{l_{\beta }} \, c}{(l_\alpha \, c + a \, \overline{c}) \, (\overline{\alpha }\, q \, c + \overline{\beta }\, a \, \overline{c})} \cdot a \, \overline{c}. \end{aligned}$$

Clearly, $\Delta _1 > 0$. Furthermore, conditions (3) and (7) imply that $\Delta _2 > 0$. This completes the proof. $\square$

## D Proof of Theorem 4

Proceeding as in the proof of Theorem 3, we calculate

$$\begin{aligned} P(\mathrm{C \vert E})= & {} \frac{l_\alpha \, c}{l_\alpha \, c + a \, \overline{c}} \\ P(\mathrm{C \vert E, \lnot O})= & {} \frac{\overline{\alpha }\, q \, c}{\overline{\alpha }\, q \, c + \overline{\beta }\,a \, \overline{c}} . \end{aligned}$$

Next, we define $\Delta _3 := P(\mathrm{C \vert E}) - P(\mathrm{C})$ and $\Delta _4 : = P(\mathrm{C \vert E, \lnot O}) - P(\mathrm{C \vert E})$ and obtain afte some algebra:

$$\begin{aligned} \Delta _3= & {} \frac{c \, \overline{c}}{l_\alpha \, c + a \, \overline{c}} \cdot (l_\alpha -a) \\ \Delta _4= & {} \frac{a \, c \, \overline{c}}{(l_\alpha \, c + a \, \overline{c})(\overline{\alpha }\, q \, c + \overline{\beta }\,a \, \overline{c})} \cdot \left( \alpha \, \overline{\beta }\, (q-p) + (\beta - \alpha ) \, q \right) \end{aligned}$$

Conditions (3) and (7) ensure that $\Delta _4 > 0$. Furthermore, $\Delta _3 > 0$ if $l_\alpha = P(\mathrm{H \vert C}) > a$. Note that $l_\alpha = P(\mathrm{H})$ for $\alpha = \beta$. Theorem 4 is therefore consistent with Theorem 2. This completes the proof. $\square$

## Footnotes

1 In this paper, we *do not* adopt reliabilism about knowledge or justification (Goldman 1967), and we do not aspire to advance current reliabilist accounts of justified credence (Dunn 2015; Tang 2016; Pettigrew 2020). Our proposal is consistent with both internalist and externalist accounts of justification.

2 Epistemologists are divided about whether epistemic akrasia is possible or rational, i. e., a case where an agent holds a credence but is not confident that they are rational in holding this credence. However, this is not the case we consider here: we imagine a case where the agent holds a credence and also assigns a reliability to herself regarding that credence. We call this the agent’s “confidence” but it is not the epistemologists’ “confidence” that designates higher-order credence or certainty.

3 Imagine a somewhat different case: instead of assigning the precise credence of 0.7 to the claim that masks lower the risk of coronavirus transmission, you assign a *range* of 0.6 to 0.8 to that same claim. Your confidence about the former might differ greatly from the confidence about the latter. For example, you might be extremely confident that your credence falls within the range indicated but not at all confident that it has the precise value of 0.7. We model this as your ascribing a high reliability to yourself regarding the range of credence but a low reliability to yourself concerning the precise number. Thus, our approach is neither committed nor restricted to cases with precise credences. However, for simplicity’s sake, we focus on the latter in the present paper. Examining confidence for ranges of credences is a topic worthy of future research. We thank an anonymous reviewer for bringing this to our attention.

4 For a survey of different models of source reliability (including the Bovens and Hartmann model) and what motivates them, see Merdes et al. (2020). This paper also references literature that provides information on empirical tests of the models in question. Note that the Bovens and Hartmann model models the reliability of an agent as a first-order probability.

5 Of course, this is but one suggestion of how confidence could be modeled. There may well be other, perhaps better, suggestions. For example, one might wish to draw on the literature on higher-order probabilities to model confidence as a second-order probability about first-order credence. See, e.g., Baron (1987), Hansson (2008) and Sahlin (1983). We thank an anonymous reviewer for this suggestion. Inspired by the success of Bovens’ and Hartmann’s model, however, we believe it is worthwhile to examine the modification of this model in more detail and to explore its consequences.

6 One might worry that this modification has an absurd result, namely having to suppose that an agent is either completely reliable, i. e., a truth-teller, or completely unreliable, i. e., entirely erratic. This would be absurd because agents are hardly ever one or the other. However, it is crucial to note that we are not committed to this assumption. This is because we do not *equate* confidence with the binary state of being either completely reliable or completely unreliable. Instead, we model confidence as a number ranging between those two extremes. An agent could well be, say, 50% confident. According to our model, they regard themselves as in-between a truth-teller and entirely erratic. We thank two anonymous reviewers for helping us to note and address this issue.

7 If *C* and *O* are positively correlated, a different ordering of the respective probabilities obtains. This would imply that the more confident the agent is, the *more* they expect serious objections from their interlocutors. We do not think that this is very plausible and therefore set aside this possibility.

8 We thank an anonymous reviewer for raising this worry.
